# The Quality and Content of Internet-Based Information on Orthopaedic Sports Medicine Requires Improvement: A Systematic Review

**DOI:** 10.1016/j.asmr.2021.05.007

**Published:** 2021-07-17

**Authors:** Ilona Schwarz, Darby A. Houck, John W. Belk, Jack Hop, Jonathan T. Bravman, Eric McCarty

**Affiliations:** Division of Sports Medicine and Shoulder Surgery, Department of Orthopedics, University of Colorado School of Medicine, Aurora, Colorado, U.S.A.

## Abstract

**Purpose:**

To evaluate the quality and content of internet-based information available for some of the most common orthopaedic sports medicine terms.

**Methods:**

A search of the PubMed, Embase, and Cochrane databases following PRISMA (Preferred Reporting Items for Systematic Reviews and Meta-analyses) guidelines was performed. All English-language literature published from 2010 to 2020 discussing information quality pertaining to orthopaedic sports medicine terms was included. Outcomes included the search engines used, number and type of websites evaluated, platform, and quality scoring metrics. Descriptive statistics are presented.

**Results:**

This review includes 21 studies. Of these, 3 evaluated both the upper and lower extremity. Twelve focused on either the upper or lower extremity, most commonly rotator cuff tears (3 of 12) and/or anterior cruciate ligament pathologies (7 of 12). The most common engines were Google (18 of 21), Bing (16 of 21), Yahoo (16 of 21), YouTube (3 of 21), Ask (3 of 21), and AOL (2 of 21). The average number of media files assessed per study was 87 ± 55. Website quality was assessed with DISCERN (7 of 21), Flesch-Kincaid (9 of 21), Health on the Net (7 of 21), and/or *Journal of the American Medical Association* Benchmark (7 of 21) scores. YouTube was evaluated with *Journal of the American Medical Association* Benchmark scores (1.74 ± 1.00). Image quality was reported in 2 studies and varied with search terminology.

**Conclusions:**

The results of this systematic review suggest that physicians should improve the quality of online information and encourage patients to access credible sources when conducting their own research.

**Clinical Relevance:**

Doctors can and should play an active role in closing the gap between the level of health literacy of their patients and that of most common online resources.

Patients have immediate access to powerful search engines and often use the internet to obtain inexpensive, quick medical advice. Previous studies have evaluated the reliability of public-access websites and have reported that many lack high-quality, accurate information.^1^

A unique subset of patients who have yet to be investigated in this context is orthopaedic athletes. Surgical interventions often have recovery periods that impact quality of life—especially in an active population in which an injury results in a significant decrease in daily activity. It is common for the surgeon to encourage limited use of an injured area or even complete immobilization to promote healing. Many active individuals facing such downtime turn to the internet since it is a wealth of information that is easy to access.

The purpose of this study was to evaluate the quality and content of internet-based information available for some of the most common orthopaedic sports medicine terms.^2^ We hypothesized that websites with a Health on the Net (HON) seal or those authored by academic institutions would provide the most medically accurate, safe, and pertinent information whereas websites published by individuals or for-profit businesses would provide the least.

## Methods

Two independent reviewers (D.A.H. and J.W.B.) searched PubMed, Embase, and the Cochrane Library up to June 12, 2020. The following search terms were used: (internet information quality) AND (anterior cruciate ligament) or (meniscal) or (shoulder instability) or (Bankart) or (rotator cuff) or (shoulder) or (tennis elbow) or (lateral epicondylitis) or (medial collateral ligament) or (posterior cruciate ligament) or (osteochondral defect) or (cartilage defect) or clavicle or knee. A total of 324 records were identified through the search of the 3 databases.

Preliminary searches were reviewed by title and/or abstract to determine study eligibility based on the inclusion criteria: studies discussing searching internet information quality pertaining to common sports medicine orthopaedic topics including anterior cruciate ligament (ACL) rupture, medial collateral ligament (MCL) tear, posterior cruciate ligament tear, meniscal tear, osteochondral defect of the knee (cartilage defect of the knee), shoulder labral tear (Bankart tear), rotator cuff tear, shoulder arthritis, clavicle fracture, and/or lateral epicondylitis (tennis elbow); full-text studies published in the English language; studies of Level I to IV evidence; and studies published from 2010 to 2020.

**Fig 1:**
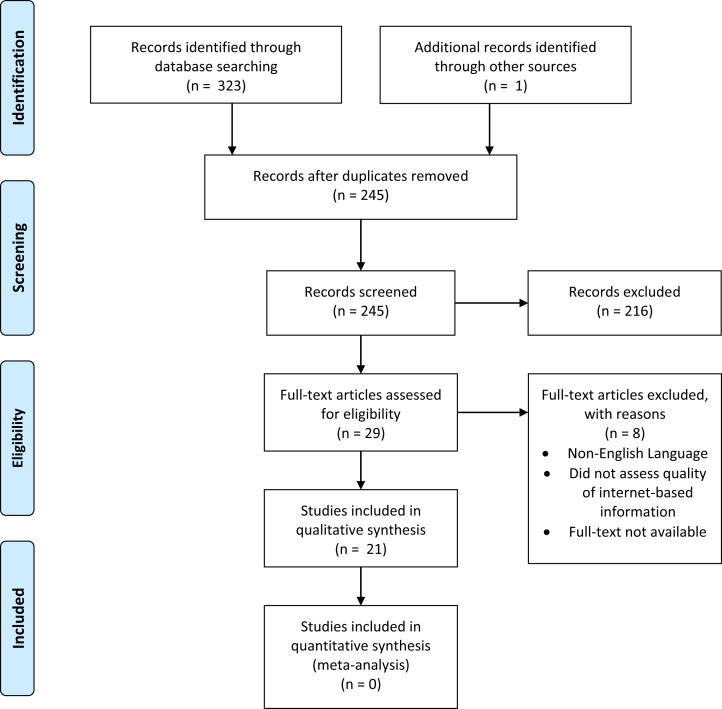
PRISMA (Preferred Reporting Items for Systematic Reviews and Meta-analyses) flow diagram showing study search and screening results.

Studies were included if they discussed searching at least one of the following databases: Google (Google LLC, Mountain View, CA), Yahoo (Verizon Media, New York City, NY), YouTube (Google LLC), Ask (IAC Search and Media, Oakland, CA), AOL (Verizon Media, New York City, NY), and/or Bing (Microsoft Corporation, Richmond, WA). Non–English-language studies, studies for which the full text was not available, cadaveric studies, basic science articles, case reports, personal correspondence, studies that did not evaluate search engines or consider a medical problem, studies that were not related to orthopaedic sports medicine, and personal correspondence were excluded. Twenty-one studies met the inclusion and exclusion criteria (Fig 1). Data extraction from each study was performed independently (I.S.). Disclosure of funding and third-party involvement were not required to obtain any of the collected data.

### Reporting Outcomes

The outcomes extracted included the primary search engines used, the number of websites evaluated by each study, the type or classification of the websites, the primary platform of the search (websites/Web pages, videos, or images), and the metrics used to score the websites. Scoring systems included the following: DISCERN instrument,^4^ Flesch-Kincaid (FK) tool,^5^, ^6^, ^7^ *Journal of the American Medical Association* (JAMA) Benchmark scores, and/or HON foundational principles.^8^, ^9^, ^10^

The DISCERN instrument consists of 15 separate questions aimed at a specific quality criterion plus an overall quality rating.^4^^,^^11^ The DISCERN categories include reliability, treatment choices, and overall quality.

The FK tool is the most widely used measure of reading ease. The tool has 2 parts: reading ease and grade level. The first number in a score indicates reading ease (0-100). The second number indicates the average reading grade level. The national average reading level is an eighth-grade level. The recommended published reading level for the layperson is a sixth-grade level.^5^ Both reading ease and the grade level are calculated using the same set of metrics: word length and sentence length. Reading ease and grade level are inversely related—a higher reading ease level correlates to a lower grade level. (Formulas are available in Appendix 1.)

The HON seal is granted based on 6 core principles: quality, confidentiality, neutrality, transparency, community, and visibility.^8^, ^9^, ^10^ The JAMA Benchmark score ranges from 0 to 4 points.^9^ The 4 criteria include author description, references, dating, and disclosures. One point is given for each of the aforementioned aspects; a score of 3 or greater is considered “high quality.”

### Statistical Analyses

Descriptive data are presented. Owing to the heterogeneity among studies, no calculable data or meta-analyses are presented in this review.

## Results

This systematic review was conducted based on the PRISMA (Preferred Reporting Items for Systematic Reviews and Meta-analyses) checklist and guidelines.^3^

### Included Studies

A total of 324 records were identified through the search of the 3 databases. Of these studies, 21 met the inclusion criteria.^4^^,^^11^, ^12^, ^13^, ^14^, ^15^, ^16^, ^17^, ^18^, ^19^, ^20^, ^21^, ^22^, ^23^, ^24^, ^25^, ^26^, ^27^, ^28^, ^29^, ^30^ Table 1 details the 21 included studies published between 2010 and 2020 that met the inclusion criteria.^5^^,^^12^, ^13^, ^14^, ^15^, ^16^, ^17^, ^18^, ^19^, ^20^, ^21^, ^22^, ^23^, ^24^, ^25^, ^26^, ^27^, ^28^, ^29^, ^30^, ^31^ All of the studies were descriptive or evaluative given the nature of the topic being discussed (Table 1).

**Table 1:** Summary of Included Studies

Study Authors	Study Title	Journal	Country	Level of Evidence
Akinleye et al.,^12^ 2018	“Readability of the Most Commonly Accessed Arthroscopy-Related Online Patient Education Materials”	*Arthroscopy*	United States	Level VI: descriptive study
Akpolat and Kurdal,^13^ 2020	“Is Quality of YouTube Content on Bankart Lesion and Its Surgical Treatment Adequate?”	*Orthopedic Surgery and Research*	Turkey	Level VI: descriptive study
Bruce-Brand et al.,^14^ 2013	“Assessment of the Quality and Content of Information on Anterior Cruciate Ligament Reconstruction on the Internet”	*Arthroscopy*	Ireland	Level VI: descriptive study
Cassidy et al.,^15^ 2018	“YouTube Provides Poor Information Regarding Anterior Cruciate Ligament Injury and Reconstruction”	*Knee Surgery, Sports Traumatology, Arthroscopy*	Ireland	Level VI: descriptive study
Celik et al.,^16^ 2020	“Assessment of the Quality and Reliability of the Information on Rotator Cuff Repair on YouTube”	*Orthopaedics & Traumatology, Surgery & Research*	Turkey	Level VI: descriptive study; case series
Dalton et al.,^17^ 2015	“Availability of Accessible and High-Quality Information on the Internet for Patients Regarding the Diagnosis and Management of Rotator Cuff Tears”	*Journal of Shoulder and Elbow Surgery*	Ireland	Level VI: descriptive study
DeFroda et al.,^18^ 2019	“Accuracy of Internet Images of Ligamentous Knee Injuries”	*The Physician and Sportsmedicine*	United States	Level VI: descriptive study
DeFroda et al.,^19^ 2018	“Internet Accuracy of Publicly Available Images of Meniscal Tears”	*The Physician and Sportsmedicine*	United States	Level VI: descriptive study
Devitt et al.,^20^ 2017	“Comparison of the Source and Quality of Information on the Internet Between Anterolateral Ligament Reconstruction and Anterior Cruciate Ligament Reconstruction: An Australian Experience”	*Orthopaedic Journal of Sports Medicine*	Australia	Level VI: descriptive study; cross sectional
Duncan et al.,^21^ 2013	“Evaluation of Information Available on the Internet Regarding Anterior Cruciate Ligament Reconstruction”	*Arthroscopy*	United States	Level VI: descriptive study
Dy et al.,^5^ 2012	“Does the Quality, Accuracy, and Readability of Information About Lateral Epicondylitis on the Internet Vary With the Search Term Used?”	*Hand (New York, NY)*	United States	Level VI: descriptive study
Garcia et al.,^22^ 2014	“Online Resources for Shoulder Instability: What Are Patients Reading?”	*Journal of Bone and Joint Surgery*—American volume	United States	Level VI: descriptive study
Goldenberg et al.,^23^ 2019	“Online Resources for Rotator Cuff Repair: What are Patients Reading?”	*Arthroscopy, Sports Medicine, and Rehabilitation*	United States	Level VI: descriptive study
Gosselin et al.,^24^ 2013	“Examining Internet Resources on Gender Differences in ACL Injuries: What Patients are Reading”	*The Knee*	United States	Level VI: descriptive study
Houck et al.,^25^ 2019	“Evaluation of Information Available on the Internet Regarding Reverse Total Shoulder Arthroplasty”	*Shoulder & Elbow*	United States	Level VI: descriptive study
Nwachukwu et al.,^26^ 2018	“The Quality of Online Resources Available to Patients Interested in Knee Biologic Therapies Is Poor”	*HSS Journal: The Musculoskeletal Journal of Hospital for Special Surgery.*	United States	Level VI: descriptive study
O'Neill et al.,^27^ 2014	“An Assessment of the Readability and Quality of Elective Orthopaedic Information on the Internet”	*Acta Orthopaedica Belgica*	Belgium	Level VI: descriptive study
Somerson et al.,^28^ 2018	“Quality of Internet-Based Decision Aids for Shoulder Arthritis: What Are Patients Reading?”	*BMC Musculoskeletal Disorders*	United States	Level VI: descriptive study
Starman et al.,^31^ 2010	“Quality and Content of Internet-Based Information for Ten Common Orthopaedic Sports Medicine Diagnoses”	*Journal of Bone and Joint Surgery*—American volume	United States	Level VI: descriptive study
Wang et al.,^29^ 2017	“Evaluation of the Quality, Accuracy, and Readability of Online Patient Resources for the Management of Articular Cartilage Defects”	*Cartilage*	United States	Level VI: descriptive study
Zhang et al.,^30^ 2016	“The Quality and Readability of Internet Information Regarding Clavicle Fractures”	*Journal of Orthopaedic Science*	United States	Level VI: descriptive study

### Internet Search Engines

Among the included studies, Google (86%),^5^^,^^12^^,^^14^^,^^17^, ^18^, ^19^, ^20^, ^21^, ^22^, ^23^, ^24^, ^25^, ^26^, ^27^, ^28^, ^29^, ^30^, ^31^ Bing (76%),^5^^,^^14^^,^^17^, ^18^, ^19^, ^20^, ^21^, ^22^, ^23^, ^24^, ^25^, ^26^, ^27^, ^28^, ^29^, ^30^ and Yahoo (76%)^5^^,^^14^^,^^17^^,^^19^, ^20^, ^21^, ^22^, ^23^, ^24^, ^25^, ^26^, ^27^, ^28^, ^29^^,^^31^ were the most commonly assessed search engines and were used for both website and image searches (Appendix Table 1).

### Website Media: Images and Video

##### Video

YouTube, the second most popular social media network, was the only search engine used to assess videos.^13^ Only 2 studies discussed the video medium: those of Akpolat and Kurdal^13^ and Cassidy et al.^15^ Cassidy et al. reported no correlation between the number of views and video quality or accuracy based on any scoring system.

##### Images

DeFroda et al.^18^^,^^19^ discussed the image medium. In their analysis of internet images based on knee ligament search terms, they found that the inter-rater reliability was high (Cronbach α = 0.89) for “PCL tear” (posterior cruciate ligament tear) searched on Bing and nearly equivalent (Cronbach α > 0.9) for the remainder of the search queries (ACL tear, MCL tear, and LCL [lateral collateral ligament] tear). When then compared Google with Bing, the only significant difference was in the ACL group. Bing returned a significantly greater number of correct images: 60% compared with Google’s 45% (*P* = .034). Otherwise, for MCL and LCL (lateral collateral ligament) tear searches, Google and Bing were not statistically significantly different. In their study assessing meniscal images, DeFroda et al.^19^ found that search engines displayed meniscal tears with greater than 80% accuracy but that many of the images were technical and required additional education in anatomy and physiology to understand and interpret.

### Website Affiliation

Most of the media files assessed were physician affiliated (25%), followed by news or other (15%) and industry or commercial (15%) (Appendix Table 2). Somerson et al.^28^ specifically considered source accuracy based on website type. They found that commercial websites had the most errors. When they compared academic sources with commercial sources, commercial sources had a 5 times greater chance of publishing false information. Nonprofit websites had the highest percentage of HON seals. Academic websites had the highest completeness score (19.2 ± 6.7; maximum, 49) when compared with commercial (15.2 ± 2.9), nonprofit (18.7 ± 6.8), and physician (16.6 ± 6.3) websites, indicating that even though a source may be factually correct, it could still be incomplete. This key point was highlighted by Wang et al.,^29^ who found that most websites, even if considered “high quality,” failed to distinguish between focal chondral defects and diffuse osteoarthritis, an important clinical factor in an orthopaedic setting.

### Study Scoring Systems

##### DISCERN Instrument

Seven studies reported on the DISCERN instrument.^13^, ^14^, ^15^, ^16^, ^17^^,^^20^^,^^25^ The average content-specific DISCERN score was 5.24, whereas the average non–content-specific DISCERN score was 40.55. The average FK grade level was 10.24, with scores ranging from 7.9 to 13.4. The average FK readability score was 52.94 (“fairly difficult, high school”). The average JAMA Benchmark score was 2.00. The average percentage of websites with HON certifications was 17.79%.

Akpolat and Kurdal^13^ and Cassidy et al.^15^ reported YouTube content-specific DISCERN scores (2.35 ± 0.91 and 2.30 ± 0.9, respectively). Celik et al.^16^ reported an average DISCERN score on YouTube of 30.5 ± 13.9. Dalton et al.^17^ reported DISCERN scores on Ask, Bing, Google, Yahoo, and AOL averaging 39.47 ± 11.39. Devitt et al.^20^ reported overall and content-specific DISCERN scores across Bing, Google, Yahoo, AOL, and Lycos (Brightcom Group, Hyderabad, Telangana, India) (overall scores of 37.3 ± 3.4 for anterolateral ligament reconstruction vs 54.4 ± 4.6 for ACL reconstruction, *P* < .0001; content-specific scores of 5.3 ± 1.3 vs 11.0 ± 1.5, *P* < .0001). Houck et al.^25^ reported an average content-specific DISCERN score across Bing, Google, and Yahoo of 3.4 ± 0.59.

##### FK Readability Test Tool

Nine studies reported on the FK readability test tool^5^^,^^12^^,^^17^^,^^22^^,^^24^^,^^26^^,^^27^^,^^29^^,^^30^: Akinleye et al.,^12^ Dalton et al.,^17^ Dy et al.,^5^ Garcia et al.,^22^ Gosselin et al.,^24^ Nwachukwu et al.,^26^ O'Neill et al.,^27^ Wang et al.,^29^ and Zhang et al.^30^ reported on grade level. Scores ranged from 7.9 to 13.4, with an average score of 10.24.

Akinleye et al.,^12^ Dalton et al.,^17^ Gosselin et al.,^24^ and O'Neill et al.^27^ reported on FK readability. Scores ranged from 47.40 to 54.60, with an average score of 52.94 (“fairly difficult, high school”).

##### Health on the Net

Seven studies reported on the HON foundational principles.^14^^,^^17^^,^^20^^,^^23^^,^^27^^,^^28^^,^^31^ The average percentage of websites with HON certifications was 17.79%. Two studies searched Ask.^14^^,^^17^ Seven searched both Google and Bing.^14^^,^^17^^,^^20^^,^^23^^,^^27^^,^^28^^,^^31^ Three searched Yahoo.^20^^,^^27^^,^^28^ Two searched AOL.^17^^,^^20^ One searched Lycos.^20^ None searched YouTube (Appendix Table 1).

##### JAMA Benchmark Score

Seven studies reported on the JAMA Benchmark score.^13^, ^14^, ^15^, ^16^, ^17^^,^^20^^,^^23^ The average JAMA Benchmark score was 2.00.

## Discussion

In this systematic review evaluating internet-based guidance for common orthopaedic sports medicine diagnoses, most search engines preferentially populate media that lacks appropriate scientific and medical screening; the best predictor for unbiased information was the presence of an HON seal and lack of third-party affiliation (i.e., financial incentive). The results of this review, along with findings presented by Akinleye et al.,^12^ support that the most frequently accessed websites exceed the reading-ease recommendations set by the American Medical Association and National Institutes of Health.^12^ Yet, as Zhang et al.^30^ reported, the use of more complex search terms provided websites with information of a higher reading grade level but not of higher quality.

Most of the websites that populate the internet when searching frequently used orthopaedic terms and diagnoses are not associated with an HON seal, meaning they are not approved for accuracy, completeness, or reliability. Many of the images that appear when searching clinical diagnoses do not align with the actual term used in the search. Finally, most videos available are non-educational and miss key clinical information. This inconsistency highlights that there exists great variability in the major search engines. In support of the findings of Bruce-Brand et al.,^14^ many of the studies in this review mentioned that health care information online frequently omits treatment options, such as doing nothing—a key feature in the DISCERN scoring rank, risks, and prognosis. Nonetheless, website accuracy, reading level, and the presence of an HON seal were positively correlated.^14^^,^^22^ Websites with a seal had higher overall DISCERN and JAMA Benchmark scores.^14^

This review emphasizes that there are very few checkpoints ensuring that medical information on the internet is vetted for safety and correctness. The 21 studies in this review stressed that awareness and use of search engines for health purposes are growing in popularity, but the general public lacks literacy regarding source credibility, which could lead to adverse health outcomes, delayed treatment, and potential exacerbation of a condition or injury. In summary, the findings of this systematic review suggest that physicians can mitigate the discrepancy in health literacy and internet information by taking an active role in guiding patients. Health care providers are in a unique position and can encourage the use of websites with HON seals and encourage patients to refrain from self-diagnosis and self-treatment based on the guidance of the internet.

### Future Directions

The problem of a physician having to prove or disprove a patient’s online diagnosis and presumed treatment merits continued analysis. Future studies should consider patient interaction with the internet and its impact on clinic visits, the added burden encountered by physicians, and potential correlations between internet use and physician visits.

### Limitations

In this study, only complete data available on the day of the search were analyzed. Therefore, variables outside the scope of the initial search, such as standardized methodologies (several studies used their own scoring tools to evaluate website content),^14^^,^^23^^,^^31^ direct implications for patients, and clinical care correlations, do not have data available for comparison. Only 2 studies looked at images, and both of those only focused on the knee, meaning there is a lack of information available on the shoulder and clavicle—other commonly injured parts.^18^^,^^19^

The only video streaming medium used was YouTube, which has additional commercial bias given that it is a social media platform. Plus, the specifications on the algorithm used by each specific search are not available and could significantly impact the results that appear. Additionally, we cannot definitively know all search-user characteristics, intentions, and biases when evaluating for a systematic review. There are limits to the generalizability of this study given that the major search engines analyzed (Google, Bing, Yahoo, AOL, and Ask) constantly undergo updates and changes to how they search, their advertisements and sponsors, and what is deemed relevant based on user and computer data. In fact, these changes over time are not well documented, and this could impact search results in every domain. Finally, there are no well-established tools used to rank health-based information that can be translated across all media forms: text, images, and videos—the closest certification for information vetting is an HON seal.

## Conclusions

The results of this systematic review suggest that physicians should improve the quality of online information and encourage patients to access credible sources when conducting their own research. Doctors can and should play an active role in closing the gap between the level of health literacy of their patients and that of most common online resources.

## Figures and Tables

**Appendix Table 1:** Search Engines Assessed in Included Studies

	Ask	Bing	Google	Yahoo	YouTube	Other
Study authors						
Akinleye et al.,^12^ 2018			X			
Akpolat and Kurdal,^13^ 2020					X	
Bruce-Brand et al.,^14^ 2013	X	X	X	X		
Cassidy et al.,^15^ 2018					X	
Celik et al.,^16^ 2020					X	
Dalton et al.,^17^ 2015	X	X	X	X		AOL
DeFroda et al.,^18^ 2019		X	X			
DeFroda et al.,^19^ 2018		X	X	X		
Devitt et al.,^20^ 2017		X	X	X		AOL, Lycos
Duncan et al.,^21^ 2013	X	X	X	X		
Dy et al.,^5^ 2012		X	X	X		
Garcia et al.,^22^ 2014		X	X	X		
Goldenberg et al.,^23^ 2019		X	X	X		
Gosselin et al.,^24^ 2013		X	X	X		
Houck et al.,^25^ 2019		X	X	X		
Nwachukwu et al.,^26^ 2018		X	X	X		
O'Neill et al.,^27^ 2014		X	X	X		
Somerson et al.,^28^ 2018		X	X	X		
Starman et al.,^31^ 2010			X	X		
Wang et al.,^29^ 2017		X	X	X		
Zhang et al.,^30^ 2016		X	X	X		
Total	3	16	18	16	3	3
%	14	76	86	76	14	14

**Appendix Table 2:** Type of Data Analyzed in Each Study and Category of Information Assessed

	No. of Media	Physician	Academic	Public Education, Not Physician	Blog	News or Other	Industry or Commercial
Study authors							
Akinleye et al.,^12^ 2018	50 websites	8	15	19	—	8	—
Akpolat and Kurdal,^13^ 2020	48 videos	—	—	—	—	—	—
Bruce-Brand et al.,^14^ 2013	60 websites	6	4	—	6	—	29
Cassidy et al.,^15^ 2018	39 websites	1	22		—	—	16
Celik et al.,^16^ 2020	67 videos	32	0	9	16	0	10
Dalton et al.,^17^ 2015	59 websites	26	—	—	—	31	2
DeFroda et al.,^18^ 2019	300 images	—	—	—	—	—	—
DeFroda et al.,^19^ 2018	300 images	—	—	—	—	—	—
Devitt et al.,^20^ 2017	81 websites	34	20	—	—	23	4
Duncan et al.,^21^ 2013	200 websites	36	23	20	12	104	5
Dy et al.,^5^ 2012	75 websites	36	4	—	45	—	40
Garcia et al.,^22^ 2014	82 websites	32	13	12	14	—	11
Goldenberg et al.,^23^ 2019	47 websites	Not specified					
Gosselin et al.,^24^ 2013	35 websites	3	7	2	13	8	2
Houck et al.,^25^ 2019	90 websites	32	38	7	1	11	1
Nwachukwu et al.,^26^ 2018	96 websites	57	9	5	6	14	5
O'Neill et al.,^27^ 2014	225 websites	Not specified					
Somerson et al.,^28^ 2018	49 websites	16	12	5	—	—	16
Starman et al.,^31^ 2010	154 websites	22	32	7	3	16	74
Wang et al.,^29^ 2017	53 websites	33	—	—	—	5	15
Zhang et al.,^30^ 2016	91 websites	Not specified					
Total	1,486 websites	374	199	86	116	220	230
%		25	13	6	8	15	15
Average ± SD	87.51 ± 55.17						

SD, standard deviation.

## Appendix 1

### Search Terms

The search terms were as follows: (internet information quality) AND (anterior cruciate ligament) or (meniscal) or (shoulder instability) or (Bankart) or (rotator cuff) or (shoulder) or (tennis elbow) or (lateral epicondylitis) or (medial collateral ligament) or (posterior cruciate ligament) or (osteochondral defect) or (cartilage defect) or clavicle or knee.

### Formulas

The following formulas were used:

Flesch-Kincaid reading-ease formula: 206.835 – 1.015 × (Number of Words/Number of Sentences) – 84.6 × (Number of Syllables/Number of Words)

Flesch-Kincaid grade-level formula: 0.39 × (Number of Words/Number of Sentences) + 11.8 × (Number of Syllables/Number of Words) – 15.59

## Supplementary Data

ICMJE author disclosure forms
